# Young Adults’ Perspectives on the Use of Symptom Checkers for Self-Triage and Self-Diagnosis: Qualitative Study

**DOI:** 10.2196/22637

**Published:** 2021-01-06

**Authors:** Stephanie Aboueid, Samantha Meyer, James R Wallace, Shreya Mahajan, Ashok Chaurasia

**Affiliations:** 1 School of Public Health and Health Systems University of Waterloo Waterloo, ON Canada

**Keywords:** self-assessment, symptom checkers, self-triage, self-diagnosis, young adults, digital platforms, internet, user experience, Google search

## Abstract

**Background:**

Young adults often browse the internet for self-triage and diagnosis. More sophisticated digital platforms such as symptom checkers have recently become pervasive; however, little is known about their use.

**Objective:**

The aim of this study was to understand young adults’ (18-34 years old) perspectives on the use of the Google search engine versus a symptom checker, as well as to identify the barriers and enablers for using a symptom checker for self-triage and self-diagnosis.

**Methods:**

A qualitative descriptive case study research design was used. Semistructured interviews were conducted with 24 young adults enrolled in a university in Ontario, Canada. All participants were given a clinical vignette and were asked to use a symptom checker (WebMD Symptom Checker or Babylon Health) while thinking out loud, and were asked questions regarding their experience. Interviews were audio-recorded, transcribed, and imported into the NVivo software program. Inductive thematic analysis was conducted independently by two researchers.

**Results:**

Using the Google search engine was perceived to be faster and more customizable (ie, ability to enter symptoms freely in the search engine) than a symptom checker; however, a symptom checker was perceived to be useful for a more personalized assessment. After having used a symptom checker, most of the participants believed that the platform needed improvement in the areas of accuracy, security and privacy, and medical jargon used. Given these limitations, most participants believed that symptom checkers could be more useful for self-triage than for self-diagnosis. Interestingly, more than half of the participants were not aware of symptom checkers prior to this study and most believed that this lack of awareness about the existence of symptom checkers hindered their use.

**Conclusions:**

Awareness related to the existence of symptom checkers and their integration into the health care system are required to maximize benefits related to these platforms. Addressing the barriers identified in this study is likely to increase the acceptance and use of symptom checkers by young adults.

## Introduction

Seeking online health information through search engines is common [1,2]; however, it can have negative effects on individuals due to the lack of reliable information and lack of health literacy or expertise of those seeking health information [3,4]. In addition to information overload, navigating the internet can be problematic due to the use of overly technical language as well as the high volume of irrelevant content returned from search engine results, the confusing layout of many web pages, and the lack of quality requirements for publishing online content [5,6]. These limitations coupled with a shortage in the health workforce globally [7] have led to the development of novel digital platforms that allow users to self-assess their symptoms. Using a question-and-answer chat format, symptom checkers prompt users to enter their symptoms and health information based on the level of care required, and a potential list of diagnoses is provided [8]. These functions allow users to self-triage (ie, assess whether they should seek medical services based on the severity of symptoms) or self-diagnose (ie, identify a health condition in oneself). Despite the mounting number of symptom checkers available and the adoption of this technology by various credible health institutions and entities such as the UK National Health Service (NHS) and the government of Australia [9,10], knowledge surrounding this technology is limited [11]. The scarce literature on symptom checker accuracy suggests that the quality of diagnostic and triage advice differs based on the digital platform used [12] with those enabled by artificial intelligence having a higher percentage of listing the correct diagnosis first [13].

In assessing symptom checker benefits, it is important to understand user perspectives on the platform after actual use rather than simply assessing their accuracy in fictitious situations [14,15]. A study that examined patients’ experiences using the symptom checker “Isabel” found that the platform was most commonly used to better understand the causes of symptoms, followed by deciding whether or not to seek care [14]. Most of the patients in that study (274/304, 90.1%) reported receiving useful information for their health problems and reported that they would use the symptom checker again (278/304, 91.4%) [14]. These findings are in line with another study that examined perspectives on use of the “Ada” symptom checker, which showed that most of the participants (443/503, 88.1%) would recommend the platform to a relative [16]. An important factor that seems to influence symptom checker acceptance is age, with younger populations exhibiting higher acceptance [14,15]. A UK-based study that engaged 1071 patients found that more than 70% of individuals between the ages of 18 and 39 years would use a symptom checker as compared to only 51% aged between 55 and 69 years [15].

A common theme that emerged from most studies examining symptom checkers is the importance of gathering user perspectives on the use of the platform to enable high acceptance (and use) as well as to prevent lost investments [17]. Given that young adults (between 18 and 34 years of age) may be the user group most accepting of such technology—and thus the ideal target group—we sought to maximize acceptance and use in this population by understanding the factors that would enable or hinder its use for self-triage and self-diagnosis. Given the relatively new emergence of symptom checkers and the prevalent use of the Google search engine (Dr. Google) for assessing symptom severity, the aim of this study was to gather university students’ perspectives after having used a symptom checker on (i) using the internet’s search engine versus a symptom checker for self-triage and diagnosis, and (ii) the enablers and barriers associated with using a symptom checker for self-triage and diagnosis.

## Methods

### Study Design and Aims

This qualitative analysis represents a subset of findings that emerged from a larger mixed-methods study that seeks to understand the factors associated with the behavioral intention of using symptom checkers for self-triage and self-diagnosis. A qualitative descriptive case study research design was used and is differentiated by other research study designs by its focus on a bounded system or case [18]. In this work, the behavioral intention of using symptom checkers is the phenomenon of interest; this phenomenon is bounded by the university campus and the selection of university students as participants. Although three notable researchers have previously described case study research, this work was conducted in line with Yin’s interpretation, which focuses on methodology and adopts a postpositivist worldview [19-21]. This work is positioned in the postpositivist paradigm due to the use of theory, the collection of data to either support or refute this theory, and the changes and revisions made to the theory as findings emerged.

### Recruitment

To allow for a broad range of perspectives to be gathered, university students between the ages of 18 and 34 years across faculties in all levels of education and year of study were eligible to participate. Following ethics approval from the Research Ethics Board at the University of Waterloo (41366), university students were notified of the study through emails from the administrative assistant of their faculty; as such, the number of students who received and opened the email is unknown. Interested individuals were asked to contact the principal investigator (SA) to schedule an interview. Participants were recruited between November 2019 and May 2020. A total of 24 participants were included in the study based on a first-come, first-served basis and time of data saturation. There were no dropouts in this study. All participants were provided with an information letter prior to the interview outlining the study objectives. Informed consent was obtained from all participants. One-on-one interviews took place on the university campus or virtually through a digital university-approved platform, Whereby. All participants were provided with a Can $10 (US $7.80) coffee shop gift card as a token of appreciation for taking the time to participate in the study.

### Data Collection

The main sources of data were a preinterview questionnaire (Multimedia Appendix 1), semistructured interview protocol (Multimedia Appendix 2), protocol for the think-aloud exercise, and clinical vignette (Multimedia Appendix 3). The semistructured interview method was used because it offers flexibility to the interviewer in determining when it is appropriate to explore certain subjects in greater depth or to pose new questions that were not originally anticipated when the interview protocol was developed [22]. To provide contextual information on each participant, the preinterview questionnaire was comprised of questions related to demographics such as age and gender as well as self-perceived health [23] and four dimensions of health literacy [24], which are two validated tools that may influence participants’ perspectives on the use of symptom checkers. Self-perceived health was measured using one question that has been previously validated [23]. Four dimensions (ie, feeling understood by health care providers, actively managing my health, ability to actively engage with health care providers, and ability to find good health information) from a total of nine of the Health Literacy Questionnaire (HLQ) questions developed by Osborne et al [24] were used; this approach has been permitted by the original authors and used in practice to reduce respondent burden. Two HLQ domains assessed in this study were measured using a 4-point Likert scale, whereas the other two were measured using a 5-point scale that ranges from “cannot or always difficult” to “always easy”; a higher number indicates a higher level of agreeableness and higher level of health literacy.

To ensure that all participants were familiar with symptom checkers, they were provided with a clinical vignette and, based on a draw, were asked to use one of two web-based symptom checkers: WebMD [25] or Babylon Health [26]. These two symptom checkers were chosen based on popularity and adoption by credible institutions such as the UK NHS, respectively. Both platforms are similar in terms of their objectives and process (eg, they both allow users to enter symptoms as free text and suggest symptoms from a drop-down list); however, there are key differences, including that Babylon Health requires the user’s full name, email address, country of residence, and date of birth. Moreover, since Babylon Health probes for more information, it may take longer to complete.

After having read the clinical vignette and accessed the symptom checker, participants were guided by the first author (SA) to conduct the think-aloud exercise, which involved the participants thinking out loud while they performed a task without synthesizing or interpreting their thoughts [27]. Similar to another study [28], the clinical vignette used depicted symptoms of a disease (ie, scarlet fever) that is less common in young adults to avoid having participants rely on recent experiences during the exercise. The questions in the interview protocol were designed to answer the main objectives of this study, which included understanding how the use of symptom checkers is perceived as compared to using the Google search engine and the factors that facilitate or hinder the use of symptom checkers. The first author (SA) conducted all interviews. SA holds a Master of Science in health systems; is trained in qualitative research methods, including data collection and analysis; and was a PhD candidate in Public Health and Health Systems at the time of the study. Given that data collection and analysis were occurring concomitantly, it was possible to cease recruitment once data saturation was reached (ie, collecting more data would not reveal new information) [29], which occurred after the interview with the 20th participant.

### Data Analysis

Data analysis was conducted independently by two authors (SA and SM) using the thematic analysis steps outlined by Castleberry and Nolen [30], which consist of compiling, disassembling, reassembling, and interpreting the data. The first step of compiling consisted of importing all transcribed interviews into the NVivo software program (version 12.6.0). To get a sense of the data as a whole, all transcripts were read in their entirety. To disassemble the data, a line-by-line coding approach was used to reduce the superimposition of preconceived notions on the data. This step generated descriptive codes [29], which were then used as a tag to retrieve and categorize similar data. Given the limited literature on this topic, the coding process was highly inductive, and a codebook was developed throughout the coding process. The codebook contains all generated codes with an indication of when they should be used.

The third step of reassembling consisted of grouping the codes into main themes; in NVivo, this consists of creating nodes (themes) and child nodes (codes under those themes)—the hierarchy can contain many levels depending on the level of detail required. In this work, a hierarchy was used to represent how themes are subordinate or superordinate to each other [30]. The final step of the analysis consisted of interpreting the analyzed data as they related to the study’s overarching aim and objectives. By interpreting the data at a higher level than themes, it was possible to answer the research question and objectives. Throughout the coding process, SA and SM discussed the identified themes and resolved any discrepancies.

## Results

### Participant Information

Most of the participants had a high score on the four health domains measured (see Table 1) with the exception of two, one, and two participants who had a low score on the following health domains: feeling understood by health care providers, actively managing my health, and ability to find good health information, respectively. The think-aloud exercise took approximately 10 to 15 minutes to complete, with those who had used the Babylon Health platform taking a longer time to complete the task due to the higher number of questions asked. A total of 11 participants were familiar with symptom checkers prior to the interview, 2 of whom had used a symptom checker for the first time to assess COVID-19–related symptoms. Participants who had previously used a symptom checker learned about the platform through word of mouth or a Google search.

**Table 1:** Participant information (N=24).

Characteristics		Value
**Gender, n (%)**		
	Female	14 (58)
	Male	9 (38)
	Nonbinary	1 (4)
**Racial group, n (%)**		
	White	9 (38)
	Asian	6 (25)
	Chinese	3 (13)
	Arab	2 (8)
	Indian	2 (8)
	Black	2 (8)
**Highest level of education, n (%)**		
	High school	2 (8)
	Undergraduate degree	14 (58)
	Master’s degree	8 (33)
**Faculty, n (%)**		
	Engineering	8 (33)
	Sciences	6 (25)
	Applied health sciences	3 (13)
	Environment	3 (13)
	Arts	3 (13)
	Mathematics	1 (4)
**Self-perceived health, n (%)**		
	Excellent	2 (8)
	Very good	13 (54)
	Good	5 (21)
	Fair	4 (17)
	Poor	0 (0)
**Health literacy, mean (range)**		
	Feeling understood by health care providers^a^	2.92 (1.5-4.0)
	Actively managing my health^a^	3.05 (1.8-4.0)
	Ability to actively engage with health care providers^b^	3.64 (2.6-4.6)
	Ability to find good health information^b^	3.81 (1.8-5.0)
**Symptom checker used, n (%)**		
	WebMD	11 (46)
	Babylon Health	13 (54)

^a^Maximum possible average is 4.

^b^Maximum possible average is 5.

### Themes Related to Using a Google Search Engine Versus a Symptom Checker

#### Theme Classification

Data related to the use of a search engine or symptom checker were grouped into positive or negative themes. Positive themes suggest a desirable attribute, function, or experience related to a platform, whereas negative themes encompass themes that suggest the opposite. An overview of these themes is provided in Textbox 1. These themes were further grouped into four main themes, which are supported by participant quotes. For example, the description under the subsection “Symptom Severity and Input” includes the main findings related to themes—both positive and negative—that pertain to how symptoms may influence participant perspectives related to the Google search engine and symptom checkers.

**Overview of themes related to using a Google search engine vs a symptom checker.**

**Positive themes**

***Google search engine***

- Provides information without claiming a diagnosis
- More customizable
- Allows entry of all symptoms in the search engine

***Symptom checkers***

- More personalized
- More interactive due to chatbot feature
- Good for those who do not know how to use Google
- Straightforward design
- Easy to use
- Real-time output
- Makes the correlation between symptoms and potential conditions
- More intuitive
- More reliable
- More specific
- More structured

**Negative themes**

***Google search engine***

- Absence of chatbot feature

Symptom checkers

- Accuracy is questionable
- Limits the number of symptoms that can be inputted
- Not widely known
- Thought process of the platform is unclear
- User more vulnerable when using this platform

***Both Google search engine and symptom checkers***

- Text input is insufficient
- Suboptimal reliability

#### Symptom Severity and Input

Participants perceived the Google search engine and symptom checkers to be useful for mild symptoms; however, some perceived that using the Google search engine was faster than having to answer questions in a symptom checker: “If you’re Googling something quick then it’s easy, quick, and straightforward, you don’t have to take 10 minutes to answer all these questions…” [P2].

Positive themes related to the use of the internet mainly pertained to the perspective that a Google search engine allows users to input as many symptoms as needed, enabling a more comprehensive search of potential conditions that may be relevant to their health context. Some users also mentioned that they prefer that the platform does not claim that this is the condition they may have.

On the other hand, I think it may be easier to get accurate results on symptoms through a Google search because I can type multiple symptoms and see how they fit, I may get more garbage results but I can use my judgment to decide what is true and not true. Whereas the symptom checker has only one piece of information which is fever. The symptom checker did not give me the opportunity to put in more from what I can recall.

P11

Nonetheless, some participants mentioned that the absence of a chatbot feature in the Google search engine limits the platform’s ability to ask follow-up questions based on symptoms inputted. As such, some users who many not be able to identify all symptoms experienced may omit certain symptoms or may not elaborate on symptoms, which hinders the quality and comprehensiveness of results.

…if I were to Google my symptom, I would just put in a fever and rashes that could be a million things. But with a symptom checker, I would put in fever and it asked me for a specific temperature and other specific questions which I would not know to search on my own.

P21

#### Perceived Characteristics of Symptom Checkers

Symptom checkers were perceived by some participants to be a good option for individuals who are less proficient in using the internet for information retrieval. Some had a positive attitude toward the symptom checkers because the platform asked questions regarding age and gender, giving the impression that it is more personalized and in turn, in their perception, more accurate.

…surfing through the internet and coming through a particular diagnosis takes a lot of time although it might give you more information about other diseases that have similar symptoms, but this is not what I am looking for, I am looking for what I am suffering from. So, for which, I think a personalized software is helpful.

P2

Some participants believed that the symptom checker “had more structure,” “provided a greater level of detail,” “was more interactive,” and “was more reliable” than using the Google search engine.

So I think having that more structured approach to inputting symptoms and figuring out what is likely wrong with you would be a lot nicer for the user and the user would have more faith in the result rather than just going on Google that brings up a whole bunch of results and the user thinking that they could have anything.

P4

#### Symptom Checker Limitations

Although having a more structured approach to symptom input was favored, some participants were unable to enter all symptoms in the platform, which led them to question the accuracy and reliability of the platform; this also hindered trust toward the platform.

I feel like I don’t like the symptom checker as much because it limits the number of symptoms. I did not have the chance to mention the thing with the red bumps; it just asked me a lot of questions about the one “symptom that was bothering me the most.”

P20

There was also a sense that participants would feel more vulnerable using a symptom checker due to the more personalized nature of the questions asked. Interestingly, some participants believed that their judgment and thought process to identify potential diagnoses was superior than using a symptom checker due to lack of knowledge about how symptom checkers work.

It feels more vulnerable and personal to put my symptoms into a list or generator of some kind. It feels like I am just looking at a series of articles I feel there's more of a distance…. If I am typing in a symptom checker and it comes back at me with answers, I don’t know how it came to that conclusion and I don’t know what the process was to decide that “yes, this is what you have,” whereas if I am the one doing the analysis through a bunch of articles that I deem legitimate—whether or not they truly are legitimate—at least I know what the thought process was and I feel like I can trust that.

P6

Despite various shortcomings that were mentioned related to the use of symptom checkers, some participants believed that an important issue is the lack of awareness about the existence of the platform: “But the issue is that we don’t know about symptom checkers so making them widely available would be super helpful.” [P13]

#### Accessing Health Services as a Preferred Option

In addition, there was a consensus that consulting a primary care provider or nurse was superior than searching the internet or using a symptom checker to assess the severity of symptoms; this was especially the case when certain symptoms required a physical examination and text input was insufficient. Reliability of the Google search engine and symptom checker was also questionable and was perceived negatively by some participants.

I think seeing a provider face to face is better than both options. I feel that you can’t accurately portray all your symptoms and general health by text input. You need someone looking at you and take measurements and touch injured areas, I think that’s far superior.

P1

I think Google is a very wide platform so it’s very hard to analyze the reliability or the source. In this case, it depends on the reliability of the symptom checker as well.

P19

### Themes Related to Barriers and Enablers of Using Symptom Checkers

#### Classification of Themes

Factors that would hinder the use of a symptom checker were identified as barriers, whereas factors that would enable an individual to use a symptom checker were identified as enablers. Participants enumerated many enablers and barriers for using symptom checkers, which were mainly related to the (1) individual, (2) disease, (3) health care system, or (4) symptom checker. An overview of all identified barriers and enablers is provided in Textbox 2. Example quotes for barriers and enablers are provided in Multimedia Appendix 4.

**Overview of enablers and barriers for using symptom checkers.**

**Individual-level factors**

***Enablers:***

- Internet access
- Low health literacy
- Trust in the platform
- Younger age
- Lack of time
- Convenience
- Lack of trust in doctors
- Curiosity
- Embarrassing topic
- Increase empowerment
- Aversion to medical professionals
- Having pre-existing conditions
- Unable to discuss the topic with a health provider
- Uncertain about care required
- Worried about health of oneself

***Barriers:***

- Lack of internet access
- Low health literacy
- Lack of trust in the platform
- Low technology literacy
- Older age
- Social influence
- Not wanting to know
- Previous bad experience

**Disease-level factors**

***Enablers:***

- Mild symptoms
- A “broad category of illness”
- Symptoms can be easily described

***Barriers:***

- Severe condition
- Need for a physical examination

**Health system–level factors**

***Enablers:***

- Approved by doctors
- Lack of access to health services
- Cost of health services
- Public education
- Increased awareness
- Long wait times for health services
- Reputable organizations recommend it

***Barriers:***

- Authoritarianism in health care

**Characteristics of symptom checkers**

***Enablers:***

- Increased advertisement
- Easy interface
- Data privacy
- Free of charge
- Short to complete
- Precision
- Use of artificial intelligence
- Gamification
- Good source of information
- Integrated with an electronic health record
- Useful in identifying potential conditions
- Information about the creators of the platform
- Interactive platform
- Reliability

***Barriers:***

- Lack of awareness
- Poor design
- Asking identifiable questions
- Cost of the platform
- Time to complete
- Lack of inclusivity measures
- Lack of language options
- Lack of credibility
- Lack of human interaction
- Disclaimer
- Inability to obtain elaboration on a question
- Liability
- Concerns about using data for profits

#### Individual-Level Factors

Internet access, health literacy, trust toward the platform, technology literacy, and age were factors mentioned to be either enablers or barriers for using a symptom checker for self-triage and self-diagnosis. Younger age, internet access, high technology literacy, and trust toward the platform were perceived to enable the use of symptom checkers. Low health literacy was perceived to be both an enabler and barrier for using a symptom checker. Although some participants believed that individuals with low health literacy are more likely to use a symptom checker because they may be less critical, others perceived low health literacy to be a barrier due to inability to understand and input symptoms into the platform.

…maybe if they just did not know a ton about health in general maybe they would be less critical than me.

P11

Also, sometimes it’s hard to articulate to have the proper term of how you feel. For example, in the fever or the lymph node, you don’t know of things like that unless you have specific knowledge about it. So, it is hard for someone who does not have medical terminology to input what they have in there.

P13

#### Disease-Level Factors

Given that a disease does not define the individual, disease-related factors were considered separately. Having a “broad category of illness,” an embarrassing issue, or an issue perceived to be mild were perceived to be enablers, whereas experiencing severe symptoms and needing a physical examination were mentioned to be barriers. Participants seemed to be more willing to use a symptom checker if they were experiencing nonspecific symptoms (eg, fatigue) due to the perceived notion that a symptom checker would allow the user to narrow down on a health condition. They are also more willing to use symptom checkers for issues perceived to be “embarrassing” such as conditions related to mental health.

If something was serious, people would not want to use it, they would want to go to a doctor. Not just physically but also emotionally, I could see them go to the doctor right away.

P13

#### Health System–Level Factors

Lack of access to and cost of health services were perceived to enable the use of a symptom checker. Having the platform approved by reputable organizations and approved by doctors were also mentioned to be important factors for enabling individuals to use the platform. In contrast, some believed that some primary care providers may not be accepting of the technology, thus limiting its use.

First of all, they have to somehow not only advertise but maybe if the website is promoted by the health care organization that is reliable for people then I can make sure that the platform is trusted by an authentic organization so for sure I would use it.

P24

An important factor related to the symptom checker was advertisement. More than half of participants were not aware of symptom checkers, thus limiting their use: “But the issue is that we don’t know about symptom checkers so making them widely available would be super helpful.” [P13]

#### Characteristics of Symptom Checkers

Developing an easy interface, guaranteeing data privacy, offering the platform free of charge, and ensuring that the platform’s questionnaire is short to complete were all mentioned as potential enablers for using a symptom checker.

It sounds very interesting and it is very easy to use. Definitely I will use it again, I had a good user experience.

P24

And if it was short—I think if there were options “hey, do you want to take the shorter version and it might not be as accurate or do you want to take the longer one that will take more time but will be more accurate.” I think people want something quick but quick won’t be as accurate.

P9

The data suggest that the main barriers for using symptom checkers are the lack of transparency on how the data collected are used; some participants mentioned that they would not have an issue with the data being used by governmental institutions to improve health services but did not want their data to be used to generate profits. Although most of the participants understood the medical terms that were used by the digital platform, some believed that the average person may not understand some of the questions asked. Providing a brief description of medical terms would allow users to interact with the platform in a more informed manner.

I would not want my data to be used to anything that would harm me. I don’t know what it could be used for but if it is being used to find out the prevalence of a certain disease or whatever that is helpful for the health care system, I am fine with that but anything that would encourage the business part of it or pharmaceutical side of it or anything that is business related or goes back to making money, I would not like it.

P3

Participants also stressed the importance for the digital platform to elaborate on why certain questions were being asked. In contrast to seeing a health professional, users are unable to interject and ask the platform questions for further elaboration. Moreover, most platforms use a disclaimer that they do not provide medical advice, which undermines the platform’s credibility.

If they know not to take it seriously, they won’t feel encouraged to do the test at all. If the disclaimer says this is not really a diagnosis, then what am I doing? I should just go to the doctor.

P10

The platforms that were used during this study were in English; however, some mentioned the importance of having these platforms available in various languages to ensure that they are accessible to those who are less proficient in English. Lack of inclusivity measures does not allow persons with disabilities to use the platform and was also mentioned as a barrier to use: “…or various disabilities being able to use the screen or use computers or any type of access issues would be a problem.” [P6]

## Discussion

### Principal Results

Approximately half of the participants were not aware about the existence of symptom checkers until their participation in this study. Most of the participants preferred consulting a health professional to address their health needs rather than researching using the internet’s search engine or using a symptom checker. Nonetheless, symptom checkers were generally preferred over the internet’s search engine due to their personalized approach; however, some perceived that the latter is faster to use than having to answer questions. There was also an acknowledgment that the results provided by symptom checkers can only be as good as the data that informed them.

In sum, it was perceived that individuals who are younger, have low health literacy, and high technology literacy were more likely to use a symptom checker. Lack of time, convenience, having symptoms that were perceived to be minor or “embarrassing” were other factors that would result in the use of a symptom checker. Enablers that were related to the health system were lack of access to care, having the technology approved by credible associations, and having symptom checkers integrated as part of the public health system. Nonetheless, participants mentioned many improvements that would have to be made to the symptom checker to enable its use, including improving accuracy, ensuring that the platform is freely accessible, and ensuring privacy and anonymity. Although the participants appreciated the personalized approach of the platform, they would not want to use a symptom checker that asks too many questions or that takes too long to complete. Barriers for using symptom checkers included lack of access to the internet, medical jargon, and lack of trust. Despite the barriers and shortcomings of the platform, participants believed that symptom checkers would be useful if they were tested and validated. Some believed that these platforms must have a positive influence on their health due to the perception that these platforms are designed by medical doctors.

### Limitations

Although this study has some strengths, various limitations warrant mention. First, given that all participants had previously used the Google search engine for seeking health information, we did not ask participants to use the Google search engine during the interview, meaning that they had to rely on their previous Google search experiences to answer questions. Second, we asked the participants if they had previously used a symptom checker, but we did not ask about the frequency of use, which limited our ability to assess whether responses differed based on this potentially important factor. Third, we did not distinguish responses based on the digital platform used, as the main focus of this work was to understand perspectives on the use of symptom checkers in general; however, these perspectives may have differed if participants used another symptom checker than those used in this study (ie, WebMD or Babylon Health). In line with this, we did not examine whether participants chose the correct diagnosis based on the clinical vignette as the focus of the study was on the process of obtaining the list of diagnoses and getting participants familiar with the platform. Last but not least, the sample was comprised of highly educated individuals who were perceived to have a good health status; as such, this may limit the transferability of findings to other populations. Future studies should explore perspectives of other user groups.

### Comparison With Prior Work

Findings from this study are in line with the literature, which suggests that using the Google search engine for health information has many limitations, including the vast amount of information available and lack of quality requirements for publishing content [5,6]. Many of these limitations could be addressed by symptom checkers as these platforms are typically developed through a collaboration between developers and medical experts. As found in other studies, symptom checkers were perceived to be a useful tool for self-assessing the severity of symptoms [14,15]; however, this was mostly the case for symptoms that are perceived to be mild and for which text input is perceived to be sufficient. Most of the participants in this study were more accepting of using symptom checkers for self-triage than for self-diagnosis. Moreover, consulting a primary care provider was the favored option over using the Google search engine or a symptom checker. This finding is in line with results from a Canadian national study, which showed that while the public supports investments in artificial intelligence and technology, they do not want to see these investments occur at the expense of the health workforce [31].

This study highlights various factors associated with the use of symptom checkers that could be used as a starting point for future investigations studying the acceptance of such technology in other population groups. Lack of time and convenience were important enabling factors for using symptom checkers; these factors also explain the use of the internet’s search engine for health information [32]. An important factor that seemed to hinder the perceived credibility of symptom checkers is that most of these platforms include a disclaimer that they are not providing medical advice. Although there are legitimate and legal reasons related to this practice, it may make people wonder why they would spend time using the platform in the first place. Ensuring that health professionals are working in conjunction with the platform has been proposed previously and may be an approach to address this issue [15].

Importantly, despite the participants reporting positive aspects of the question-answer format used by the symptom checker, most would have favored a more interactive platform that provides more information to the user regarding why certain questions are being asked. Participants also mentioned the importance of being able to ask questions to the platform—this is something that could be easily done during a conversation with a medical provider or a Google search; however, given the more rigid nature of most symptom checkers, this feature is not yet readily available. This is important for symptom checker developers to consider as patient-centered communication has been shown to be important for patient outcomes [33]. Moreover, participants mentioned that they would be more trusting of this platform if it provided them with a diagnosis that they thought they had, which is in line with another study [34], indicating the presence of confirmation bias.

Lack of internet access is also a critical element that hinders access to any web-based information platform. Although lack of internet access is more prevalent in developing countries [35], it remains an issue for certain remote and rural regions in Canada. However, efforts to address this issue have been outlined in the 2019 Canadian budget in which the government announced its commitment of reaching a target of 95% of Canadian homes and businesses having access to internet speeds of at least 50/10 Mbps (50 Mbps downloading speed and 10 Mbps uploading speed) by 2026 and 100% access by 2030 [36]. Interestingly, the lack of connectivity is related to poor literacy and digital skills rather than to lack of affordability [35]. This highlights the importance of ensuring that populations have the means of accessing these platforms. Failing to do so will undermine the purpose and mission of many of these technology companies that aim to reach those that are disadvantaged and living in developing countries [37]. Participants also mentioned that lack of human interaction may be a potential barrier for older adults, but they did not believe it to be a barrier for them. The importance of integrating human support in technology has been recommended for improving adherence, communication with care teams, and improving the quality of tool use [38].

In line with perspectives from experts in the field [39], symptom checkers have the potential to improve quality in health care; however, various barriers should be addressed to improve acceptance and use of the platform by end users. Given the wide array of factors elucidated in this study, future studies should focus on understanding the relative importance of these factors as they relate to the acceptance and use of symptom checkers. For example, participants can be asked to rank the barriers and enablers for using symptom checkers in order of their perceived importance. Importantly, to ensure client-centric product development, companies or governmental institutions developing these platforms should include end users in the process. Similarly, seeking health care provider perspectives should be prioritized to inform how symptom checkers should be utilized by the health care system to maximize its benefits while ensuring that they meet user needs.

### Conclusions

Symptom checkers are promising tools and seem to be more accepted for self-triage rather than for self-diagnosis. To maximize acceptance and use among young adults, it is important to address the various barriers identified in this study, including those that seek to improve the user experience. Importantly, awareness related to the existence of symptom checkers and their integration into the health care system are required to maximize the benefits related to these platforms. Future studies targeting other group segments are needed to understand perspectives of symptom checker use among the wider population.

## Appendix

Multimedia Appendix 1 Preinterview questionnaire.

Multimedia Appendix 2 Interview questionnaire.

Multimedia Appendix 3 Think-aloud exercise protocol and clinical vignette.

Multimedia Appendix 4 Example quotes of enablers and barriers for using symptom checkers.

## Abbreviations

HLQ: Health Literacy Questionnaire
NHS: National Health Service
