# Supplementary material for: Young Adults’ Perspectives on the Use of Symptom Checkers for Self-Triage and Self-Diagnosis: Qualitative Study
Source: JMIR Public Health Surveill. 2021 Jan 6;7(1):e22637. doi: 10.2196/22637 (PMC7817365; doi:10.2196/22637)
Supplement: Multimedia Appendix 1 [file publichealth_v7i1e22637_app1.docx]

## Preinterview Questionnaire

1. **How old are you?** _______
2. **What is your self-perceived gender?**

□ Female □ Male □ Non-binary

1. **What is your self-perceived racial or cultural group?**

□ First Nations

□ White

□ South Asian (e.g., East Indian, Pakistani, Sri Lankan)

□ Chinese

□ Black

□ Filipino

□ Latin American

□ Arab

□ Southeast Asian (e.g., Vietnamese, Cambodian, Malaysian, Laotian)

□ West Asian (e.g., Iranian, Afghan)

□ Korean

□ Japanese

□ Other (please specify): ___________

1. **What is your highest level of education?**

□ High school

□ Undergraduate degree

□ Master’s

□ PhD

1. **What program are you currently enrolled in?**

□ Undergraduate degree

□ Master’s

□ PhD

□ Other (please specify): ____________

1. **In which faculty do you currently study at the University of Waterloo?**

□ Applied Health Sciences

□ Arts

□ Engineering

□ Environment

□ Mathematics

□ Science

1. **Are you currently employed?**

□ Yes

□ No

□ Prefer not to disclose

1. **If you answered yes to the question above, how many hours (on average), do you currently work within a week period?**

□ 1 – 5 hours

□ 6 – 10 hours

□ 11 – 15 hours

□ 16 – 20 hours

□ 21 – 25 hours

□ 26 – 30 hours

□ 31 – 35 hours

□ 36 – 40 hours

1. **Compared to others your own age, how would you rate your health? Please circle the option that applies.**

□ Poor

□ Fair

□ Good

□ Very good

□ Excellent

1. **Please indicate how strongly you disagree or agree with each of the following statements. Remember to check only one box for each statement.**

|  | Strongly disagree | Somewhat disagree | Somewhat agree | Strongly agree |
| --- | --- | --- | --- | --- |
| Consumers have lost all control over how personal information is collected and used by companies. |  |  |  |  |
| Most businesses handle the personal information they collect about consumers in a proper and confidential way. |  |  |  |  |
| Existing laws and organizational practices provide a reasonable level of protection for consumer privacy today. |  |  |  |  |
| I have a good understanding of technical terms such as “cookies”, “encryption”, “certificate”, “Firewall” and “WPA/2”. |  |  |  |  |
| I am familiar with the “Terms of Use” of the online services I sign up for. |  |  |  |  |
| I have a good understanding of how to use the basic functions on my smartphone. |  |  |  |  |

1. **Please indicate how strongly you disagree or agree with each of the following statements. Remember to check only one box for each statement.**

|  | Strongly disagree | Disagree | Agree | Strongly agree |
| --- | --- | --- | --- | --- |
| I have at least one healthcare provider who knows me well |  |  |  |  |
| I spend quite a lot of time actively managing my health |  |  |  |  |
| I have at least one healthcare provider I can discuss my health problems with |  |  |  |  |
| I make plans for what I need to do to be healthy |  |  |  |  |
| Despite other things in my life, I make time to be healthy |  |  |  |  |
| I have the healthcare providers I need to help me work out what I need to do |  |  |  |  |
| I set my own goals about health and fitness |  |  |  |  |
| There are things that I do regularly to make myself healthier |  |  |  |  |
| I can rely on at least one healthcare provider |  |  |  |  |

1. **Please indicate how difficult or easy the following tasks are for you now. Remember to check only one box for each statement.**

|  | **Cannot or always difficult** | **Usually difficult** | **Sometimes difficult** | **Usually easy** | **Always easy** |
| --- | --- | --- | --- | --- | --- |
| Make sure that healthcare providers understand your problems properly |  |  |  |  |  |
| Feel able to discuss your health concerns with a healthcare provider |  |  |  |  |  |
| Have good discussions about your health with doctors |  |  |  |  |  |
| Discuss things with healthcare providers until you understand all you need to |  |  |  |  |  |
| Ask healthcare providers questions to get the health information you need |  |  |  |  |  |
| Find information about health problems |  |  |  |  |  |
| Find health information from several different places |  |  |  |  |  |
| Get information about health so you are up to date with the best information |  |  |  |  |  |
| Get health information in words you understand |  |  |  |  |  |
| Get health information by yourself |  |  |  |  |  |
