# Supplementary material for: Young Adults’ Perspectives on the Use of Symptom Checkers for Self-Triage and Self-Diagnosis: Qualitative Study
Source: JMIR Public Health Surveill. 2021 Jan 6;7(1):e22637. doi: 10.2196/22637 (PMC7817365; doi:10.2196/22637)
Supplement: Multimedia Appendix 2 [file publichealth_v7i1e22637_app2.docx]

## Interview Questionnaire

**Internet and symptom checker usage**

1. What are your thoughts on the use of the internet to find health information for triage?
2. What are your thoughts on the use of the internet to find health information for self-diagnosis?

The next questions will focus on the use of symptom checkers. In this study, we define symptom checkers as digital platforms that utilize AI techniques that seek to mimic human intelligence and serve two main functions of triage and self-diagnosis based on symptoms and data inputted by users.

1. Do you currently use symptom checkers?
   - If so, how long have you used this technology?
   - How did you hear about it?
2. Based on the definition I provided, what are your perspectives on this technology?

**Barriers, facilitators, and opportunities**

1. What are your thoughts on the use of symptom checkers as compared to conducting a general search on the internet?
2. What do you believe are the enablers (factors that facilitate) for using symptom checkers?
3. What do you believe are the barriers (factors that hinder) for using symptom checkers?
4. What do you believe are the opportunities with the use of symptom checkers?
5. Did you use a symptom checker to check for symptoms related to COVID-19?
   1. Why or why not?
   2. If so, how would you describe your experience?
   3. *(If the person replies that they did not use one because they did not develop symptoms, then ask):* Would you have used one if you did develop symptoms?
6. How do you think symptom checkers can influence the health of university students?
7. How do you believe symptom checkers will influence the use of health services?

**Use of AI in health care and trust**

1. What is your outlook on the use of artificial intelligence in health care?
2. How much do you trust this technology?
3. What do you think about the output provided by the platform?
4. Would you still want to visit a primary care provider to review the diagnosis following the use of this technology?

**User experience and concerns**

1. How do you feel about having to choose one of the diagnosis provided on the platform?
2. What do you believe are the other capabilities that would make symptom checkers more useful or attractive to university students?
3. Can you tell me about any concerns you might have about using a symptom checker for triage or self-diagnosis?

**Closing Questions**

1. Do you believe that your answers would have been different if you were interviewed before the COVID-19 pandemic?
2. Is there a question you feel I should have asked but did not?
3. Is there anything you would like to add?
