# Supplementary material for: Young Adults’ Perspectives on the Use of Symptom Checkers for Self-Triage and Self-Diagnosis: Qualitative Study
Source: JMIR Public Health Surveill. 2021 Jan 6;7(1):e22637. doi: 10.2196/22637 (PMC7817365; doi:10.2196/22637)
Supplement: Multimedia Appendix 3 [file publichealth_v7i1e22637_app3.docx]

## Think-Aloud Exercise Protocol and Clinical Vignette

**Interviewer:** The first part of the interview is now complete, and we will now begin the second portion which entails a think-aloud exercise.

**Interviewer:** For the second part of the interview, we are interested in understanding the thought process of university students while they use a digital platform for triage or self-diagnosis. To complete this task, please read the following vignette and use the WebMD symptom checker (or Babylon) to enter in the relevant information and symptoms. During the process, please say out loud everything that you are thinking without synthesizing your thoughts. It will be important for you to keep talking as you perform the task.

**Interviewer provides an example of how the thinking-aloud exercise should be performed.*

**Interviewer:** Do you have any questions about this exercise? Please feel free to practice thinking out loud before starting. You can start whenever you feel comfortable.

**Participant completes the task*

**Interviewer:** I now have a few questions about the use of this platform.

**Clinical Vignette:**

I’ve been feeling sick for almost a week. I have a high fever and the lymph nodes in my neck are swollen. I also have this weird, red rash on my neck and arms. My tongue has red bumps on it too. I wish I would feel better soon.
