# Supplementary material for: Young Adults’ Perspectives on the Use of Symptom Checkers for Self-Triage and Self-Diagnosis: Qualitative Study
Source: JMIR Public Health Surveill. 2021 Jan 6;7(1):e22637. doi: 10.2196/22637 (PMC7817365; doi:10.2196/22637)
Supplement: Multimedia Appendix 4 [file publichealth_v7i1e22637_app4.docx]

## Example quotes of enablers and barriers for using symptom checkers.

| **Individual-level enablers** | |
| --- | --- |
| **Enabler** | **Example Participant Quote** |
| Internet access | “*Access to the internet*.” – P16 |
| Low health literacy | “…*maybe if they just did not know a ton about health in general maybe they would be less critical than me*.” – P11 |
| Trust in the platform | “*If you want people to use symptom checkers more, there has to be development on the technology to make people trust it*.” – P21 |
| High technology literacy | “*Health literacy and technological literacy are big ones*.” – P10 |
| Younger age | “*The younger generation would be a lot more into doing this type of thing because I know that the older generation are less savvy with using new technology and programs*.” – P2 |
| Lack of time | “*Entirely be time and lack of availability of health services. Sitting at a drop-in clinic it sucks, obviously. So, if you don’t have to go through that, it’s great – from a time perspective but also from sheer stress and not being enjoyable to sit at a clinic*.” – P1 |
| Convenience | “*Just generally wait times with family practitioners I mean it can obviously take several weeks or longer to make an appointment with a practitioner and the inconvenience of the wait for the appointment and to get to where the appointment is.*” – P6 |
| Lack of trust in doctors | “*If you don’t trust the doctor, you may still end up using the symptom checker*.” – P16 |
| Curiosity | “*Curiosity, I think people are just generally interested in what’s going to pop up based on the symptoms.*” – P4 |
| Embarrassing topic | “*Some people are embarrassed of health issues so if they can have it diagnosed without having to see someone then they would be interested in that.*” – P1 |
| Increase empowerment | “*I find it empowering to be able to identify what is going on with my own body so a symptom checker can allow me to get a sense of what might be going on – put words to something I cannot put words to*.” – P23 |
| Aversion to medical professionals | “*Perhaps an aversion to actual medical professionals. People generally don’t like doctors it seems or healthcare professionals and that might be the environment of the medical office or the personal privacy standpoint of it. That will probably continue and so I see a benefit of these programs and why they exist.*” – P4 |
| Having pre-existing conditions | “*If you have pre-existing conditions, if you know you’re generally not well and generally anxious about your health, you will go ahead and use this to check the problem*.” – P15 |
| Unable to discuss the topic with a health provider | “*Embarrassment of discussing the topic or unable to discuss it with the doctor (so either lacking the knowledge back or doctors not being trained in informing their patients properly or not taking the time to inform patients).*” – P4 |
| Uncertain about care required | “*I think people will use it when they are uncertain if something is serious and have to go to the emergency or if it’s something that they can put off and take an appointment with the doctor*.” – P13 |
| Worried about health of oneself | “*If you know you’re generally not well and generally anxious about your health, you will go ahead and use this to check the problem. Being always worried about diseases and health*.” – P16 |
| **Disease-level enablers** | |
| **Enabler** | **Example Participant Quote** |
| Mild symptoms | “*Something that is personal or lame [something minor] just to check if it’s abnormal.*” – Participant 3 |
| A “broad category of illness” | “…*if it’s still a broad category of illnesses, then they would just go to a symptom checker.*” – P2 |
| Symptoms can be easily described | “*Maybe if they have symptoms that can be easily described, and they know how to describe them properly*.” – P18 |
| **Health-system level enablers** | |
| **Enabler** | **Example Participant Quote** |
| Approved by doctors | “*Advertised by a doctor, not advertised but you know in the doctor’s office where you have some papers and ads so having that there so it’s at the doctor’s and you have more confidence that it will be something useful and not just weird things that go on the internet*.” – P13 |
| Lack of access to health services | “*Entirely time and lack of availability of health services.*” – P1 |
| Cost of health services | “*I think that maybe if they had to pay for healthcare wherever they are they would try to figure it out if it’s actually serious*.” – P11 |
| Public education | “*Educating the public is very important because the older generation probably doesn’t trust the internet stuff. I would trust it a bit more, but I am still hesitant because I do not know how this thing works and anything that deals with your health, people are a bit more cautious about it*.” – P21 |
| Increased awareness | “*But I would say a very attractive platform and ads to make it known that these things exist*.” – P3 |
| Long wait times for health services | “*Just generally wait times with family practitioners I mean it can obviously take several weeks or longer to make an appointment with a practitioner and the inconvenience of the wait for the appointment and to get to where the appointment is*.” – P6 |
| Reputable organizations recommend it | “*First of all, they have to somehow not only advertise but maybe if the website is promoted by the healthcare organization that is reliable for people then I can make sure that the platform is trusted by an authentic organization so for sure I would use it, why not*.” – P24 |
| **Symptom checker related enablers** | |
| **Enabler** | **Example Participant Quote** |
| Increased advertisement | “*But I would say a very attractive platform and ads to make it known that these things exist. Especially during cold and flu season if there are advertisements in bathroom stalls or cafeterias.*” – P3 |
| Easy interface | “*It sounds very interesting and it is very easy to use. Definitely I will use it again, I had a good user experience*.” – P24 |
| Data privacy | “*They will use it if they know that their information is not going to be shared. These days people are very hung up on keeping their information private. To all their own but a lot of people do not feel comfortable putting their name and email address when they ask for it before you start or before you get your result.*” – P10 |
| Free of charge | “*Cost, in the United States you have to pay to see a doctor and why pay if you can have something that may give you a better diagnosis or idea of what is going on.*” – P8 |
| Good source of information | “*Something that I found really weird about health information sharing is that one of the key messages that you receive in anything health related is “talk to your doctor” but my experience has been that talking to my doctor, I would be talking to my doctor all the time. So, I feel that symptom checkers or other sources of health information that people have access to and can trust can help people get good healthcare information without constantly having to take an appointment and go to the medical office*.” – P23 |
| Short to complete | “*And if it was short – I think if there were options “hey, do you want to take the shorter version and it might not be as accurate or do you want to take the longer one that will take more time but will be more accurate”. I think people want something quick but quick won’t be as accurate.*” – P9 |
| Precision | “*This is a precise and confidential type of platform where you just have to put it in your age and gender*.” – P17 |
| Use of AI | “*Maybe it would seem more comforting that there is an AI behind it rather than just Googling*.” – P20 |
| Gamification | “… *there’s probably a gamification way to help people use it too – maybe a monthly check-in thing where you do a personal assessment and you get points or badges for being on top of your own health. I feel like I would use that more if I had that*.” – P6 |
| Integrated with an electronic health record | “*I think if the symptom checker was somehow able to send a report of the symptoms to your doctor, I think that would help*.” – P8 |
| Useful in identifying potential conditions | “*So, this might be kind of a slightly less intense less scary less vulnerable less committed way of digging what condition it might be*.” – P6 |
| Information about the creators of the platform | “*The experience of the practitioners who developed this tool and that have practiced for many years*.” – P19 |
| Interactive platform | “*Some sort of interface that could be more user friendly. This is user friendly, but it should be a bit more interactive and should allow to ask questions*.” – P19 |
| Reliability | “*I think if the symptom checkers are from sources which are reliable which again is a dicey statement to make because someone could find something reliable or not*.” – P19 |

| **Individual-level barriers** | |
| --- | --- |
| **Barrier** | **Example Participant Quote** |
| Lack of internet access | “*Also, if we are talking about people who do not have access. Even though it’s not as common as before, you still have people who don’t have access to the internet or know that this service is possible*.” – P9 |
| Low health literacy | “*Also, sometimes it’s hard to articulate to have the proper term of how you feel. For example, in the fever or the lymph node, you don’t know of things like that unless you have specific knowledge about it. So, it is hard for someone who does not have medical terminology to input what they have in there*.” – P13 |
| Lack of trust in the platform | “*People might not want to piss off their doctors or have a general distrust of things on the Internet. You never know who is putting out really bad information out there*.” – P12 |
| Low technology literacy | “*If you don’t know how to use technology properly – elderly people may not know how to – I taught my grandmother how to use chrome – some people just don’t know how to use the internet. Some people don’t know how to google properly – they will google a full sentence rather than googling ‘symptom checker’.” –* P8 |
| Older age | “*I know my grandma who has a lot of illnesses and medication does not use the internet, so she does not even google anything*.” – P2 |
| Social influence | “*Family telling you not to use it because of previous bad experiences*.” – P16 |
| Not wanting to know | “… *just fear or avoidance – not wanting to know*.” – P6 |
| Previous bad experience | “*Misdiagnosis or a previous bad experience*.” – P16 |
| **Disease-level barriers** | |
| **Barrier** | **Example Participant Quote** |
| Severe condition | “*If something was serious, people would not want to use it, they would want to go to a doctor. Not just physically but also emotionally, I could see them go to the doctor right away*.” – P13 |
| Need for a physical examination | “*Can only be used for symptoms that can be described in words. I feel like this can act as a barrier for skin problems because it needs an assessment (physical one).*” – P17 |
| **Health-system level barriers** | |
| **Barrier** | **Example Participant Quote** |
| Authoritarianism in healthcare | “*A lot of dogma and a lot of doctors will say ‘we are always the right ones, do not access these tools". A lot of doctors are like that, they will say ‘don’t bother googling your symptoms just come and see me’ and other doctors really appreciate that people try to educate themselves with whatever tool they have to their disposal which is often Google or a tool like this*.” – P10 |
| **Symptom checker related barriers** | |
| **Barrier** | **Example Participant Quote** |
| Lack of awareness | “*Lack of knowledge – there are people who do not have an idea that there is service like this which gives results fast*.” – P16 |
| Poor design | “*Lack of user-friendly interface. The UX [user experience] was terrible, it was really frustrating. Too much information especially for someone who does not have the medical knowledge*.” – P20 |
| Asking identifiable questions | “*I might think ‘Oh, what are they using my data for?’. It depends, as I said, I have not used a symptom checker before, so I don’t know what kind of information they are asking for. I wouldn’t mind giving them my age and gender but for example, if they are asking for date of birth and things like that, that might give a red flag*.” – P3 |
| Cost of the platform | “*Also cost might be a barrier*.” – P9 |
| Time to complete | “*I think another barrier is time depending on how long it can be. I am sure it can vary*.” – P2 |
| Lack of inclusivity measures | “*[…] or various disabilities being able to use the screen or use computers or any type of access issues would be a problem*.” – P6 |
| Lack of language options | “*Maybe also a barrier can be the language that is used in the software*.” – P2 |
| Lack of credibility | “*Lack of credibility*.” – P18 |
| Lack of human interaction | “*The human interaction and I know we are getting away from that because the way technology is designed, we don’t have to talk to people in the grocery store anymore to buy our groceries so it’s something we are getting more socialized to. But for seniors it’s a barrier because of the trust component and human interaction but also technology literacy and I think that would also apply to people with disabilities who still need to see someone face to face to get a better idea of the issues*.” – P7 |
| Disclaimer | “*If they know not to take it seriously, they won’t feel encouraged to do the test at all. If the disclaimer says this is not really a diagnosis, then what am I doing? I should just go to the doctor*.” – P10 |
| Inability to obtain elaboration on a question | “*I think it’s the inability to get an elaboration on something. If you are talking to a person you can ask them questions and they hopefully elaborate on it and tell you what that means but with a symptom checker the onus is on you to go further*.” – P4 |
| Liability | “*Liability. Like if it was jumping to severe and worst-case scenarios*.” – P1 |
| Concerns about using data for profits | “*I would not want my data to be used to anything that would harm me. I don’t know what it could be used for but if it is being used to find out the prevalence of a certain disease or whatever that is helpful for the healthcare system, I am fine with that but anything that would encourage the business part of it or pharmaceutical side of it or anything that is business related or goes back to making money, I would not like it*.” – P3 |
